# Towards Unravelling the Role of ERα-Targeting miRNAs in the Exosome-Mediated Transferring of the Hormone Resistance

**DOI:** 10.3390/molecules26216661

**Published:** 2021-11-03

**Authors:** Olga E. Andreeva, Danila V. Sorokin, Ekaterina I. Mikhaevich, Irina V. Bure, Yuri Y. Shchegolev, Marina V. Nemtsova, Margarita V. Gudkova, Alexander M. Scherbakov, Mikhail A. Krasil’nikov

**Affiliations:** 1 Department of Experimental Tumour Biology, Institute of Carcinogenesis, N.N. Blokhin National Medical Research Center of Oncology of the Ministry of Health of the Russian Federation, 115522 Moscow, Russia; o.andreeva@ronc.ru (O.E.A.); d.sorokin@ronc.ru (D.V.S.); k.mihaevich@gmail.com (E.I.M.); yurashhegolev@gmail.com (Y.Y.S.); gudkova@ronc.ru (M.V.G.); krasilnikovm1@yandex.ru (M.A.K.); 2 Laboratory of Medical Genetics, Institute of Molecular Medicine, I.M. Sechenov First Moscow State Medical University, 119991 Moscow, Russia; bureira@mail.ru (I.V.B.); nemtsova_m_v@mail.ru (M.V.N.)

**Keywords:** signalling pathways, tamoxifen resistance, exosomes, miRNA (microRNA), breast cancer, oestrogen receptor, ESR1, SERM, miR-181a-2

## Abstract

Hormone therapy is one of the most effective breast cancer treatments, however, its application is limited by the progression of hormonal resistance, both primary or acquired. The development of hormonal resistance is caused either by an irreversible block of hormonal signalling (suppression of the activity or synthesis of hormone receptors), or by activation of oestrogen-independent signalling pathways. Recently the effect of exosome-mediated intercellular transfer of hormonal resistance was revealed, however, the molecular mechanism of this effect is still unknown. Here, the role of exosomal miRNAs (microRNAs) in the transferring of hormonal resistance in breast cancer cells has been studied. The methods used in the work include extraction, purification and RNAseq of miRNAs, transfection of miRNA mimetics, immunoblotting, reporter analysis and the MTT test. Using MCF7 breast cancer cells and MCF7/T tamoxifen-resistant sub-line, we have found that some miRNAs, suppressors of oestrogen receptor signalling, are overexpressed in the exosomes of the resistant breast cancer cells. The multiple (but not single) transfection of one of the identified miRNA, miR-181a-2, into oestrogen-dependent MCF7 cells induced the irreversible tamoxifen resistance associated with the continuous block of the oestrogen receptor signalling and the activation of PI3K/Akt pathway. We suppose that the miRNAs-ERα suppressors may act as trigger agents inducing the block of oestrogen receptor signalling and breast cancer cell transition to an aggressive oestrogen-independent state.

## 1. Introduction

In recent years, many new classes of antitumour compounds with a significant effect on the signalling pathways in tumour cells have been developed [1,2,3,4,5,6]. Along with the new classes, antihormonal drugs—SERMs (selective oestrogen receptor modulators) and SERDs (selective oestrogen receptor degraders)—remain highly relevant as an antitumour therapy [7,8,9]. Hormone therapy [10,11,12,13,14] is one of the most common types of treatment of hormone-dependent tumours including breast cancer, ovarian cancer, endometrial and prostate tumours. Hormone therapy is based on the principle of creating an artificial deficiency of hormones necessary for the growth of hormone-dependent tumours, oestrogens (for tumours of the female reproductive system) and androgens (for prostate tumours). This effect is achieved mainly in two ways: by reducing the concentration of endogenous hormones, due to the inhibition of their synthesis (aromatase inhibitors), or replacing hormones with their inactive analogues (antioestrogens or antiandrogens).

According to the World Health Organization, there were more than 2.2 million women diagnosed with breast cancer and 685,000 deaths worldwide in 2020. There are about eight million women alive who have been diagnosed with breast cancer in the last 5 years. Breast cancer is the most common cancer in the world and about 70% of breast tumours contain oestrogen receptors (ERα) [10,15,16,17]. Tumours with HER2/neu expression belong to the HER2-positive group. Tumours that do not contain steroid hormone receptors and HER2 are classified as triple-negative cancers. The oestrogen receptor α status of breast cancer is a predictive parameter of the response to hormone therapy [18,19]. Developed in the 60s, tamoxifen (2-[4-[(*Z*)-1,2-diphenylbut-1-enyl]phenoxy]-*N*,*N*-dimethylethanamine), an antihormonal agent belonging to SERMs, has become widespread [20,21,22,23]. These days, tamoxifen remains the “gold standard” for hormonal therapy. It is important to note that tamoxifen can be prescribed for a long period. Ten years of tamoxifen treatment reduced the incidence of breast cancer recurrence by more than 25%, compared with 5 years of tamoxifen treatment [24]. Despite the high effectiveness of tamoxifen, the problem of resistance to this drug is especially urgent [7,25,26,27].

As a rule, the development of tamoxifen resistance is caused either by an irreversible block of hormonal signalling (suppression of the activity or synthesis of specific intracellular hormone receptors) or by activation of growth-regulating signalling pathways that bypass hormone-dependent signalling. So, in addition to a receptor depletion, among the main factors promoting hormonal resistance, one can distinguish an imbalance between activator proteins and receptor suppressors, ligand-independent activation of the receptor, stimulation of hormone-independent signalling pathways (primarily tyrosine kinase receptors) and thereby support of tumour growth in the absence of hormones [28,29,30,31,32,33,34,35]. One of the best-known examples of such activation is overexpression of Her2/neu oncogene in breast cancer cells, a member of the tyrosine kinase receptor family that controls cell proliferation in the absence of oestrogens [36,37,38]. In addition, signalling proteins overexpressed in resistant cells include proteins that suppress the activity of the oestrogen receptor (nuclear factor kappa B (NF-κB)) or prevent ligand-dependent receptor activation (phosphatidylinositol-4,5-bisphosphate 3-kinase/AKT serine/threonine kinase (PI3K/Akt), p21 (RAC1) activated kinase 1 (PAK1)) [39,40,41,42]; Snail and other regulators of epithelial-mesenchymal transition (EMT) [43,44].

Namely, PI3K/Akt signalling belongs to the key anti-apoptotic pathways that determine the cancer cell defence against anti-tumour treatment. The constitutive activation of PI3K/Akt signalling was found in many types of tumours, including breast and prostate tumours, ovarian, uteri tumours etc. Being the central player in the transferring of mitogen stimuli, PI3K/Akt signalling is involved in the compensatory overcoming of the drug-induced growth block [45,46,47]. Similarly, the hormonal resistance of tumours including breast cancer is often accompanied by the activation of PI3K/Akt signalling; the recent observations demonstrate its direct involvement in the maintaining of the hormone-resistant phenotype of tumours [48,49,50,51].

The participation of miRNAs in the development of hormonal resistance in tumours has been demonstrated quite convincingly. These are, first of all, miRNAs involved in the negative regulation of the oestrogen receptor: miR-221/222 [52] miR-342 [53], Let7b/Let-7i [54], miR-1280 [55], miR-873 [56] and some others. In addition, miRNAs are involved in the regulation of oestrogen signalling, the targets of which are the oestrogen receptor coactivator/corepressor proteins, in particular, miR-17-5p, which regulates the expression of the steroid receptor coactivator protein 3 (SRC-3) [57], miR-10 targeting the nuclear co-repressor NCOR2 [58]; miR-451, which regulates the expression of the signalling proteins HER2 (human epidermal growth factor receptor 2), EGFR (epidermal growth factor receptor), and MAPK (mitogen-activated protein kinase) [59], miR-101, which is involved in the regulation of Akt signalling in resistant cells [60]. Among tumour suppressor proteins, PTEN (phosphatase and tensin homolog) holds a special place, as it is a target for several miRNAs associated with hormonal resistance [61,62,63]. EMT proteins are also among the targets of miRNAs involved in antioestrogen resistance: increased expression of miR-205 driven by Mel-18 protein downregulates zinc finger E-box binding homeobox proteins ZEB1 and ZEB2 and therefore restores E-cadherin expression in breast cancer cells and xenografts [64]. The suppression of miR-7, targeting ABC drug efflux pump MRP1 (multidrug resistance-associated protein 1), was found to promote multidrug resistance in breast cancer and other cancer types [65].

Recent data demonstrate the possibility of the horizontal transfer of hormonal resistance (cell-to-cell) [66,67,68,69]. Extracellular vesicles play a critical role in this process due to the exosomal miRNAs, among which miRNAs have been identified that are involved in the negative regulation of the oestrogen receptor: miR-342, Let7b / Let-7i, miR-1280 and others [70]. Delivered into the target cell, such miRNAs can induce a rearrangement or blockage of hormonal signalling and, as a consequence, a decrease in the sensitivity to hormone therapy. Since miRNAs are expressed and/or delivered to a cell with exosomes in the form of a “rich cocktail” containing hundreds of miRNAs, it is extremely important to identify exosomal miRNAs associated with the hormonal resistance and to determine their ability to induce the irreversible rearrangement of oestrogen signalling.

## 2. Results

Experiments were performed on the in-vitro-cultured oestrogen-dependent MCF7 cells and tamoxifen-resistant MCF7/T subline obtained by long-term tamoxifen treatment of the parent cells. Earlier we have shown that long-term treatment of the MCF7 cells with the exosomes from the resistant MCF7/T cells results in the development of tamoxifen resistance in the recipient cells [71]. To further the study of the mechanism of the exosome-induced resistance, the profile of exosomal miRNAs was analysed. We proposed that continuous incorporation of the miRNA–suppressors of the oestrogen receptor (ERα) into the cells results in the irreversible block of the oestrogen signalling and compensatory activation of the oestrogen-independent growth pathways forming the tamoxifen-resistant phenotype.

MiRNA profile of the MCF7 and MCF7/T exosomes was studied, and miRNAs overexpressed in the resistant cells and exosomes were identified (Supplementary data, Table S1). Furthermore, we have searched for associations between identified miRNAs and ESR1 using the integrative database of human miRNA target predictions mirDIP, and six miRNAs overexpressed in the exosomes of resistant cells were annotated as potentially targeting ERα and suppressors of oestrogen signalling (Table 1). Among them, miR-181a-2 was overexpressed in both resistant cells and respective exosomes (Table S1). Moreover, miR-181a-2 was found to be within 1% of top miRNAs annotated with ESR1 (very high confidence class) supporting the possible involvement of the latter in the acquisition of hormonal resistance.

**Figure 1:**
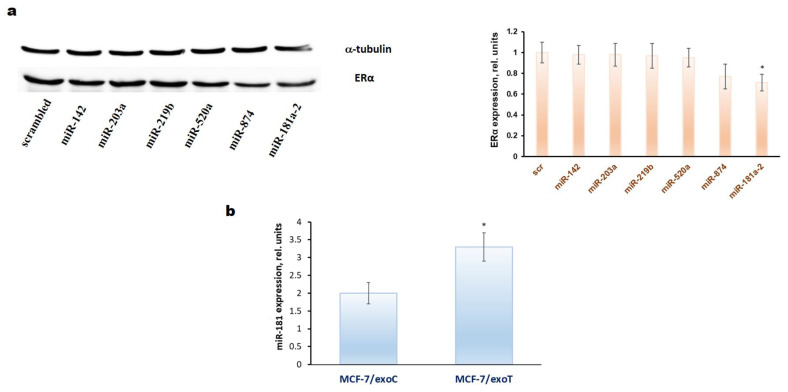
(**a**) Influence of miRNAs transfection on the ERα expression in the MCF7 cells. The single transfection by the miRNAs mimetics was performed as described in Methods. Twenty-four hours after transfection the Western blot analysis of ERα expression in the cell lysates was performed. Protein loading was controlled by membrane hybridization with α-tubulin antibodies. Densitometry for immunoblotting data (right diagram) was carried out using ImageJ software (Wayne Rasband, NIH) with the recommendations from the work [83]; * *p* < 0.05 versus scrambled (scr); (**b**) quantification of endogenous miR-181a expression (vertical axis) in the exosome-treated MCF-7 cells by qRT-PCR. The MCF-7 cells were cultured in the presence of the exosomes isolated from MCF-7 (exoC) and MCF-7/T (exoT) cells for 30 days with following cell cultivation within the next 30 days after exosomes withdrawal. Three separate measurements were performed for each sample. The expression of RNU6B was used as an internal control. Error bars indicate standard deviation; * *p* < 0.05 versus MCF-7 cells treated with exoC.

The comparative analysis of the identified miRNAs’ influence on the ERα expression showed that miR-181a-2 demonstrates the maximal inhibitory activity (Figure 1a), and all following experiments were performed with the latter. The role of this miRNA in cancer progression is of great interest as its expression correlates with cell proliferation and with the survival of cancer patients [79,80,81].

Using PCR analysis of miR-181a-2 content in the exosome-treated cells we have revealed the constitutively increased level of miR-181a-2 in the MCF-7 cells treated with the MCF-7/T exosomes demonstrating the pivotal role of exosomal miR-181a-2 in the transferring of the resistance (Figure 1b). The full protocol of the exosome preparation and development of exosome-induced resistance was described previously [82].

**Figure 2:**
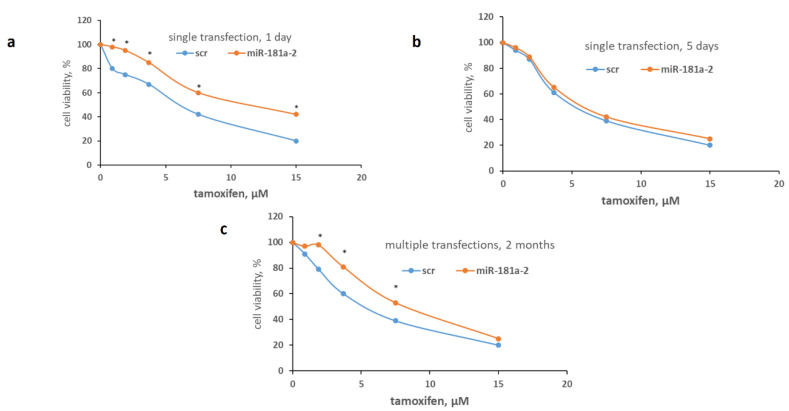
Influence of the miR-181a-2 on the cell sensitivity to tamoxifen. The single (**a**,**b**) or multiple (**c**) transfection of MCF7 cells with miR-181a-2 mimetic or scrambled RNA construct as control was performed as described in Methods. To determine the cell sensitivity to tamoxifen the cells in one day (**a**), five days (**b**) after single transfection or the cells cultured for two months after multiple transfections (**c**) were treated with tamoxifen for 72 h and the number of the viable cells was counted by the MTT test. Data represent the mean value of three independent experiments. * *p* < 0.05 versus scrambled (scr).

We have found that single transfection of miR-181a-2 into MCF7 cells results in temporary tamoxifen resistance being decreased in 5 days after transfection. On the contrary, multiple transfections (totally, 20 rounds of transfection) of miR-181a-2 induce the persistent tamoxifen resistance, and transfected cells retain the resistance to tamoxifen within at least 2 months of cultivation after the last transfection (Figure 2).

**Figure 3:**
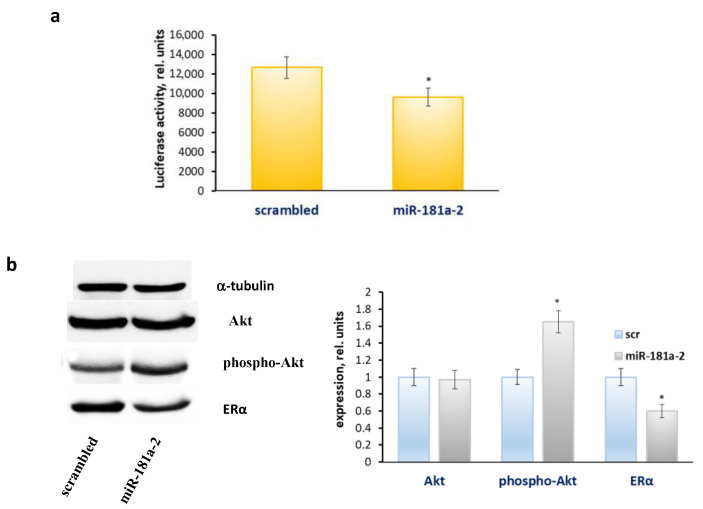
The effect of the multiple transfections of miR-181a-2 on the ERα transcriptional activity and protein profile. The MCF7 cells were cultured for two months after multiple transfections with miR-181a-2-mimetic or scrambled-RNA construct, then the cells were transfected with the ERE plasmid containing the luciferase reporter gene under the oestrogen-responsive element (ERE) and β-galactosidase plasmid (**a**). The relative luciferase activity was calculated in arbitrary units as the ratio of the luciferase to the galactosidase activity. Data represent the mean value ± SD of three independent experiments. The viability of cells treated with vehicle control was set at 100%. (**b**) Western blot analysis. The MCF7 cells were treated as indicated above. Western blot analysis of Akt, phospho-Akt and ERα was performed in the MCF7 cell extracts. Protein loading was controlled by membrane hybridization with α-tubulin antibodies. Densitometry for immunoblotting data (right diagram) was carried out using ImageJ software; * *p* < 0.05 versus scrambled (scr).

Furthermore, the transfected cells were characterized by the sustained suppression of the ERα level and its transcriptional activity accompanied by the constitutive activation of Akt (Figure 3).

**Figure 4:**
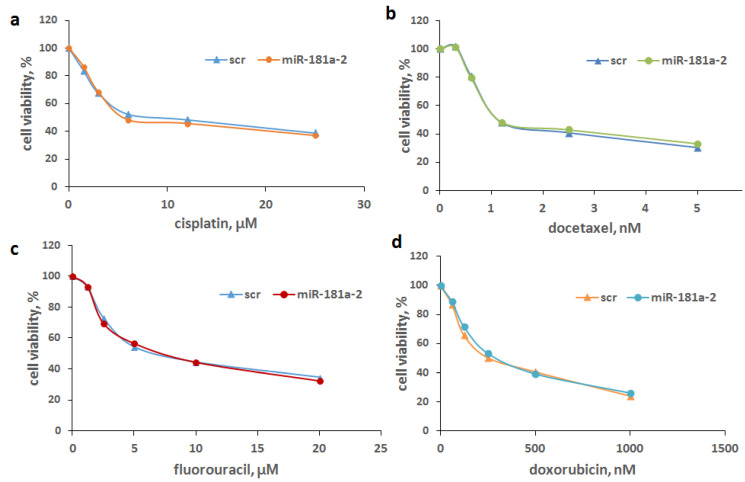
Drug sensitivity of the miR-181a-2-multiple transfected cells. The multiple-transfected MCF7 cells were obtained as indicated above. The cell sensitivity to cisplatin (**a**), docetaxel (**b**), fluorouracil (**c**) and doxorubicin (**d**) was determined by the MTT test. Data represent the mean value ± SD of three independent experiments.

The analysis of the cell sensitivity to nonhormonal cytostatic drugs, cisplatin, docetaxel, fluorouracil and doxorubicin, revealed no difference in the sensitivity of the miR-181a-transfected cells and control cells to these drugs demonstrating the high specificity of the acquired tamoxifen resistance in these cells (Figure 4).

**Figure 5:**
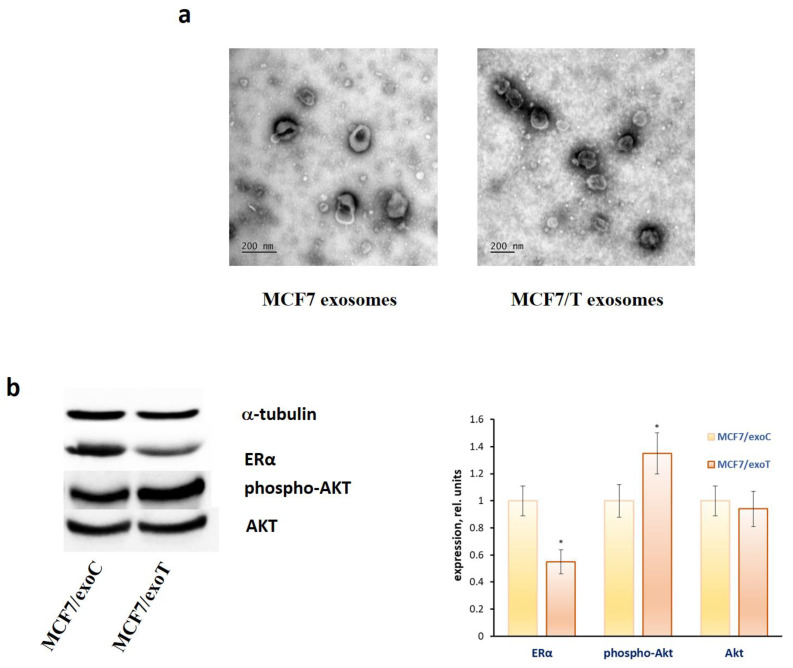
Exosome influence on the ERα and Akt level in MCF7 cells. (**a**) The transmission electron microscopy of the exosomes. Exosomes were collected from the MCF7 and MCF7/T conditioned medium by the differential ultracentrifugation, labelled by the gold nanoparticles and imaged as described in Methods. (**b**) ERα and Akt expression. MCF7 cells were treated with exosomes from MCF7 and MCF7/T for 1 month and Western blot analysis of ERα and Akt was performed in the cell extracts. Protein loading was controlled by membrane hybridization with α-tubulin antibodies. Densitometry for immunoblotting data (right diagram) was carried out using ImageJ software; * *p* < 0.05 versus scrambled (scr).

As mentioned above, the exosomes of the resistant MCF-7/T cells are characterized with overexpression of miR-181a-2 (Table S1). To compare the effects of miR-181a-2 and exosomes produced by the parent or resistant cells the analysis of the oestrogen signalling in the exosome-treated cells was performed. Exosomes were isolated from the MCF7 and MCF7/T conditioned medium by the differential ultracentrifugation, and exosome imaging was carried out by transmission electron microscope as described in Methods (Figure 5a). The recipient MCF7 cells were cultured in the presence of exosomes from MCF7 or resistant MCF7/T cells in the final concentration of 1.7 μg/mL for 1 month and oestrogen and Akt signalling was analysed. We have revealed the same tendency: suppression of ERα expression and activation of Akt phosphorylation in the cells treated with the exosomes from the MCF7/T-resistant cells (Figure 5b).

## 3. Discussion

The antioestrogen resistance of breast cancer cells can be achieved in multiple ways [34,35,84,85,86]. It can be accompanied by induction of antiapoptotic cascades (downregulation of proapoptotic Bax, suppression of PARP (poly [ADP-ribose] polymerase) and caspase-3 cleavage), cell cycle progression (Cyclin D1) and the activation of Akt-mediated Wnt signalling [87]. Different factors are affecting the switch of signalling cascades targeted on tamoxifen resistance-associated genes (NRIP1 (nuclear receptor-interacting protein 1), CCND1 (Cyclin D1), IGFBP4, IGFBP5 (insulin-like growth factor binding proteins)). In the work [88], for instance, it was shown that protein RBP2 (retinol-binding protein 2) can activate the ER-IGF1R-ErbB signalling cascade to induce tamoxifen resistance.

Recently many miRNAs have been found to be associated with the progression of tamoxifen resistance of breast cancer cells [89,90,91], however, the miRNAs involvement in the resistance development is still unclear. Here we have found that some miRNAs–suppressors of oestrogen signalling are overexpressed in the exosomes of the tamoxifen-resistant cells. Considering the possible involvement of exosomes in the transferring of hormonal resistance [32,92], the role of some of these miRNAs in the progression of acquired resistance was analysed.

Many researchers point to the important role of the miRNA axis in the progression of various cancers [93,94,95,96]. It is now clear that miRNAs could be evaluated as predictors of the response to therapy and as biomarkers. Aiko Sueta and colleagues revealed that a combined signature of four miRNAs (miR-4448, miR-2392, miR-2467-3p and miR-4800-3p) could be used to discriminate between pCR and non-pCR patients with triple-negative breast cancer [97]. It is interesting to note that not only intracellular miRNAs, but also circulating miRNAs, have great potential for use as potent biomarkers [98]. Intriguing new data suggest that miRNAs will be used to overcome resistance. Bernice Monchusi and Mandeep Kaur suggested that targeting the miRNAs hsa-miR-34a-5p and hsa-miR-373-3p could provide a way to alter the cell’s response to drugs via modulating cholesterol pathways in cancer stem cells [99].

As revealed, the multiple (but not single) transfection of miR-181a-2 into oestrogen-dependent MCF7 cells induces the irreversible tamoxifen resistance demonstrating the important role of this miRNA in the formation of the resistant phenotype. We suppose that prolonged cell treatment with the miRs-ERα suppressors induces the continuous block of the oestrogen signalling resulting in the progression of the hormone resistance. Probably, the mechanism of such resistance may be similar to that induced by prolonged antioestrogen treatment—in both cases, the central event includes the prolonged suppression of oestrogen receptor with the following cell switch to oestrogen-independent growth. In agreement with the latter, we have revealed the constitutive activation of Akt in the cells after multiple transfections of miR-181a-2 showing the involvement of PI3K/Akt signalling in the growth regulation of the resistant cells. The miRs-ERα suppressors are overexpressed in the exosomes of the resistant cells, allowing us to consider such miRs as one of the key factors involved in the progression of exosome-induced resistance. The results obtained are in agreement with the recent publications demonstrating the ability of the exosomes of tamoxifen-resistant cells to induce resistance in the recipient cells [100,101]. Here we have revealed oestrogen receptor machinery as one of the possible targets of exosomal miRNAs transferring the resistant phenotype in breast cancer cells.

## 4. Conclusions

Presently, we have demonstrated that miR-181a-2 has the ability to downregulate ERα expression in the oestrogen-dependent MCF-7 breast cancer cells. Analysis of the profile of exosomal miRs in the MCF-7 cells and resistant MCF-7/T subline revealed the overexpression of miR-181a-2 in the exosomes of the resistant cells. We have shown that regular treatment of the MCF-7 cells with the resistant exosomes as well as the multiple (but not single) transfection of miR-181a-2 results in the progression of tamoxifen resistance in the treated cells accompanied with the constitutive activation of PI3K signalling. In general, we suggest that ERα-targeting miRNAs may be involved in the transferring of the hormonal resistance via the prolonged blockage of oestrogen signalling accompanied with the activation of oestrogen-independent pathways.

## 5. Materials and Methods

### 5.1. Cell Lines

The human breast cancer cell line MCF7 was purchased from ATCC. The cells were cultured in a standard DMEM medium (Gibco (Thermo Fisher Scientific, Waltham, MA, USA)) supplemented with 10% foetal bovine serum (FBS) (HyClone (Cytiva, Marlborough, MA, USA)) at 37 °C and 5% CO_2_. The tamoxifen-resistant MCF7/T subline was established from the parent MCF7 cells by long-term tamoxifen treatment, as described [66].

### 5.2. Exosome Isolation and Visualization

Exosomes were prepared from the MCF7 and MCF7/T conditioned medium by the differential ultracentrifugation and were characterized as described in our recent paper [102]. Transmission electron microscopy (TEM) with immunogold labelling was used to visualize the exosome samples. Imaging was carried out using a JEM-1011 (JEOL Ltd., Tokyo, Japan) transmission electron microscope at 80 kV. At least 30 images were obtained for the exosomes of each type.

### 5.3. MiRNA Analysis

The analysis of exosomal miRNAs was performed by HiSeq2500 (Illumina, San Diego, CA, USA) and at least 5 million reads per sample were obtained. Library preparation and sequencing was done by ZAO Genoanalytica as follows: miRNA was extracted from exosomes by PureLink RNA Micro Kit (12183-016 (Thermo Fisher Scientific, Waltham, MA, USA)) according to the manual. Library preparation was carried out with the NEBNext Small RNA Library Prep Set for Illumina (E7330S (New England Biolabs, Hitchin, UK)). The associations between miRNAs and ESR1 were searched by using the integrative database of human miRNA target predictions mirDIP [103], which aggregates the data from all known miRNA databases. The likelihood level was specified as medium. All studied miRNAs were found as potentially targeting ESR1. Some of them (miR-181a-2-3p and hsa-miR-874-3p) were found to be within 1% of top miRNAs annotated with ESR1 (very high confidence class). Moreover, these 6 miRNAs were selected concerning the literature indicated in Table 1. All hyperexpressed miRNAs are provided in Table S1 in the supporting file.

### 5.4. RNA Isolation and Quantification by Quantitative RT–PCR

Total RNA was extracted using TRIzol reagent (Life Technologies, Carlsbad, CA, USA) by a phenol-chloroform extraction method [82]. The quantity and purity of extracted RNA were measured with the NanoDrop-1000 spectrophotometer (Thermo Fisher Scientific, Waltham, MA, USA). cDNA synthesis was carried out with 1 μg total RNA by using the MiScript Reverse Transcription Kit (Qiagen, Hilden, Germany). Quantitative reverse transcription-polymerase chain reactions (qRT- PCR) for miR-181a-2 and housekeeping gene RNU6B were performed in triplicates of 12 μL by using the CFX96 Touch Real-Time PCR Detection System (Bio-Rad, Hercules, CA, USA) and the MiScript SYBR Green PCR Kit (Qiagen, Hilden, Germany). PCR primer sequence for miR-181a-2 was: 5′- CACTGACCGTTGACTGTACC -3′. Pre-synthesized primer for RNU6B was used (Hs_ RNU6B_13 (miScript Primer Assay (Qiagen, Hilden, Germany)).

### 5.5. MiRNA Transfection

MiRNA constructs were purchased from Syntol. RNAs were dissolved in annealing buffer (10 mM Tris-HCl, pH 7.5, 50 mM NaCl, 1 mM EDTA) at 100 µM concentration and annealed at room temperature following heating to 95 °C. Transient single or multiple transfections of miRNAs were performed using Lipofectamine 2000 (Thermo Fisher Scientific, Waltham, MA, USA) to result in the final RNA concentration of 50 nM. Multiple transfections were performed twenty times, once every three days.

### 5.6. Immunoblotting, Reporter Analysis, and MTT-Test

Protein expression was determined by immunoblotting [104], reporter analysis used to study the transcriptional activity of oestrogen receptor (ERα) was described in our work [105]. Cell viability was assessed by the MTT test as described in [106]. All in vitro experiments were performed in triplicates. The Student’s t-test was used to evaluate the significance of differences in comparisons. The *p*-value of <0.05 was considered statistically significant.

## Figures and Tables

**Table 1:** List of the studied miRNAs and their functions.

MicroRNAs	Biological Activity	Reference
142	targets ESR1, reduces cell viability, induces apoptosis and decreases colony formation	[72]
203a	is overexpressed in breast cancer and can influence ERα signalling by targeting ADCY5, IGF1, etc.	[73]
219b	downregulates ERα	[74]
520a	targets NF-κB and TGF-β signalling pathways; targets ESR1, inhibits CCND1 mRNA and cyclin D1 protein levels	[75,76]
874	targets ESR1, CDK9	[77]
181a	downregulates ERα; induces AKT signalling	[74,78]

## Data Availability

Data available from the authors.

## Supplementary Materials

The following is available online, Table S1. miRNAs hyperexpressed in tamoxifen-resistant cells and their exosomes (in comparison with miRNAs from control exosomes/cells).
