# Supplementary material for: Towards Unravelling the Role of ERα-Targeting miRNAs in the Exosome-Mediated Transferring of the Hormone Resistance
Source: Molecules. 2021 Nov 3;26(21):6661. doi: 10.3390/molecules26216661 (PMC8588049; doi:10.3390/molecules26216661)
Supplement: Supplementary file 1 [file molecules-26-06661-s001.zip › molecules-1434165-supplementary.pdf]

Supporting information

Towards Unravelling the Role of ERα-Targeting miRNAs in the Exosome-Mediated Transferring of the Hormone Resistance

Olga E. Andreeva [1], Danila V. Sorokin [1], Ekaterina I. Mikhaevich [1], Irina V. Bure [2], Yuri Y. Shchegolev [1], Marina V. Nemtsova [2], Margarita V. Gudkova [1], Alexander M. Scherbakov [1,*] and Mikhail A. Krasil'nikov [1]

1 Department of Experimental Tumour Biology, Institute of Carcinogenesis, N.N. Blokhin National Medical Research Center of Oncology of the Ministry of Health of the Russian Federation, 115522 Moscow, Russia; o.andreeva@ronc.ru (O.E.A.); d.sorokin@ronc.ru (D.V.S.); k.mihaevich@gmail.com (E.I.M.); yurashhegolev@gmail.com (Y.Y.S.); gudkova@ronc.ru (M.V.G.); krasilnikovm1@yandex.ru (M.A.K.)

2 Laboratory of Medical Genetics, Institute of Molecular Medicine, I.M. Sechenov First Moscow State Medical University, 119991 Moscow, Russia; bureira@mail.ru (I.V.B.); nemtsova_m_v@mail.ru (M.V.N.)

* Correspondence: a.sherbakov@ronc.ru or alex.scherbakov@gmail.com

Table S1. MiRNAs hyperexpressed in tamoxifen-resistant cells and their exosomes (in comparison with miRNAs from control exosomes/cells). The **miR-181a-2** entries are highlighted in green.

miRNAs Hyperexpressed in Exosomes from Tamoxifen-Resistant Cells	miRNAs Hyperexpressed in Tamoxifen-Resistant Cells
hsa-miR-5196-3p	hsa-miR-12136
hsa-miR-150-5p	hsa-miR-200b-5p
hsa-miR-6815-5p	hsa-miR-200a-5p
hsa-miR-598-5p	hsa-miR-34a-5p
hsa-miR-4321	hsa-miR-3605-5p
hsa-miR-1224-5p	hsa-miR-30c-5p
hsa-miR-1247-5p	hsa-miR-30c-1-3p
hsa-miR-4783-3p	hsa-miR-186-5p
hsa-miR-6892-5p	hsa-miR-197-3p
hsa-miR-3661	hsa-miR-197-5p
hsa-miR-940	hsa-miR-320b
hsa-miR-6818-3p	hsa-miR-9-5p
hsa-miR-6781-3p	hsa-miR-556-5p
hsa-miR-4540	hsa-miR-181b-5p
hsa-miR-1246	hsa-miR-181b-3p
hsa-miR-425-5p	hsa-miR-181a-5p
hsa-miR-4700-3p	hsa-miR-181a-3p
hsa-miR-3606-3p	hsa-miR-29b-3p
hsa-miR-3651	hsa-miR-664a-3p
hsa-miR-1247-3p	hsa-miR-3155b
hsa-miR-6075	hsa-miR-4676-3p
hsa-miR-4784	hsa-miR-146b-5p
hsa-miR-4431	hsa-miR-2110
hsa-miR-6506-3p	hsa-miR-378c

hsa-miR-4763-5p	hsa-miR-210-3p
hsa-miR-1251-3p	hsa-miR-1237-3p
hsa-miR-4285	hsa-miR-139-3p
hsa-miR-4445-3p	hsa-miR-708-5p
hsa-miR-6876-3p	hsa-miR-708-3p
hsa-miR-4682	hsa-miR-141-5p
hsa-miR-579-3p	hsa-miR-1293
hsa-miR-187-5p	hsa-miR-196a-5p
hsa-miR-509-3p	hsa-miR-618
hsa-miR-4632-3p	hsa-miR-3059-5p
hsa-miR-6731-3p	hsa-miR-331-5p
hsa-miR-1245b-5p	hsa-miR-331-3p
hsa-miR-124-5p	hsa-miR-15a-5p
hsa-miR-619-5p	hsa-miR-17-5p
hsa-miR-25-5p	hsa-miR-17-3p
hsa-miR-329-3p	hsa-miR-20a-5p
hsa-miR-1915-5p	hsa-miR-625-5p
hsa-miR-4290	hsa-miR-625-3p
hsa-miR-607	hsa-miR-337-3p
hsa-miR-146b-3p	hsa-miR-431-3p
hsa-miR-548as-3p	hsa-miR-379-5p
hsa-miR-3940-3p	hsa-miR-381-3p
hsa-miR-4469	hsa-miR-655-3p
hsa-miR-4695-3p	hsa-miR-134-5p
hsa-miR-1292-5p	hsa-miR-1266-5p
hsa-miR-656-3p	hsa-miR-629-5p
hsa-miR-199a-5p	hsa-miR-629-3p
hsa-miR-4733-5p	hsa-miR-1276
hsa-miR-4762-5p	hsa-miR-3177-3p
hsa-miR-4771	hsa-miR-193b-5p
hsa-miR-1237-5p	hsa-miR-193b-3p
hsa-miR-6790-3p	hsa-miR-365a-3p
hsa-miR-127-3p	hsa-miR-365a-5p
hsa-miR-203a-3p	hsa-miR-6511a-5p
hsa-miR-1295a	hsa-miR-6770-3p
hsa-miR-154-5p	hsa-miR-4721
hsa-miR-3180-5p	hsa-miR-140-3p
hsa-miR-193b-5p	hsa-miR-3182
hsa-miR-4638-3p	hsa-miR-22-5p
hsa-miR-5688	hsa-miR-22-3p
hsa-miR-574-3p	hsa-miR-324-5p
hsa-miR-7107-3p	hsa-miR-324-3p
hsa-miR-193b-3p	hsa-miR-4521
hsa-miR-6769b-3p	hsa-miR-744-3p
hsa-miR-6819-5p	hsa-miR-33b-3p
hsa-miR-4689	hsa-miR-193a-5p
hsa-miR-6501-3p	hsa-miR-193a-3p
hsa-miR-5006-5p	hsa-miR-365b-3p

hsa-miR-6071	hsa-miR-152-3p
hsa-miR-4426	hsa-miR-6785-5p
hsa-miR-3613-3p	hsa-miR-6516-5p
hsa-miR-7847-3p	hsa-miR-99b-3p
hsa-miR-4485-3p	hsa-let-7e-5p
hsa-miR-4654	hsa-miR-125a-3p
hsa-miR-23a-5p	hsa-miR-3127-3p
hsa-miR-203b-5p	hsa-miR-128-3p
hsa-miR-4791	hsa-miR-128-1-5p
hsa-miR-1225-3p	hsa-miR-1246
hsa-miR-1254	hsa-miR-5001-5p
hsa-miR-6757-5p	hsa-miR-663a
hsa-miR-7973	hsa-miR-548o-3p
hsa-miR-1470	hsa-miR-1914-5p
hsa-miR-1343-3p	hsa-miR-185-5p
hsa-miR-378f	hsa-miR-1306-5p
hsa-miR-4651	hsa-miR-301b-3p
hsa-miR-6865-3p	hsa-miR-130b-5p
hsa-miR-7113-3p	hsa-miR-130b-3p
hsa-miR-548aw	hsa-miR-3200-3p
hsa-miR-6840-3p	hsa-miR-3909
hsa-miR-4417	hsa-miR-33a-3p
hsa-miR-766-3p	hsa-miR-548ay-3p
hsa-miR-6861-5p	hsa-miR-26a-1-3p
hsa-miR-3141	hsa-miR-1226-5p
hsa-miR-6803-3p	hsa-miR-7976
hsa-miR-6511b-3p	hsa-miR-573
hsa-miR-4486	hsa-miR-574-3p
hsa-miR-761	hsa-miR-4449
hsa-miR-4800-5p	hsa-miR-576-5p
hsa-miR-412-3p	hsa-miR-3139
hsa-miR-151a-5p	hsa-miR-579-5p
hsa-miR-6512-3p	hsa-miR-143-3p
hsa-miR-4754	hsa-miR-340-3p
hsa-miR-323a-5p	hsa-miR-3691-5p
hsa-miR-219a-1-3p	hsa-miR-3143
hsa-miR-4729	hsa-miR-877-5p
hsa-miR-2277-3p	hsa-miR-4640-5p
hsa-miR-645	hsa-miR-3934-5p
hsa-miR-6797-3p	hsa-miR-1275
hsa-miR-501-5p	hsa-miR-7111-3p
hsa-miR-378j	hsa-miR-30c-2-3p
hsa-miR-7977	hsa-miR-30a-5p
hsa-miR-498	hsa-miR-30a-3p
hsa-miR-3184-5p	hsa-miR-589-5p
hsa-miR-492	hsa-miR-590-3p
hsa-miR-506-5p	hsa-miR-10525-3p
hsa-miR-7110-3p	hsa-miR-653-5p

hsa-miR-4659a-3p	hsa-miR-653-3p
hsa-miR-325	hsa-miR-4652-5p
hsa-miR-933	hsa-miR-597-3p
hsa-miR-4787-3p	hsa-miR-320a-3p
hsa-miR-3940-5p	hsa-miR-6847-5p
hsa-miR-4690-5p	hsa-miR-491-5p
hsa-miR-4756-3p	hsa-let-7f-1-3p
hsa-miR-1208	hsa-miR-181a-2-3p
hsa-miR-7152-3p	hsa-miR-3911
hsa-miR-554	hsa-miR-4669
hsa-miR-6763-5p	hsa-miR-532-5p
hsa-miR-6798-5p	hsa-miR-500b-5p
hsa-miR-1291	hsa-miR-660-5p
hsa-miR-181a-2-3p	hsa-miR-98-3p
hsa-miR-210-5p	hsa-let-7f-2-3p
hsa-miR-767-5p	hsa-miR-421
hsa-miR-6088	hsa-miR-374a-3p
hsa-miR-27b-5p	hsa-miR-361-3p
hsa-miR-4731-3p	hsa-miR-652-3p
hsa-miR-1249-5p	hsa-miR-652-5p
hsa-miR-1910-5p	hsa-miR-450b-5p
hsa-miR-612	hsa-miR-450a-5p
hsa-miR-874-5p	hsa-miR-542-3p
hsa-miR-6889-5p	hsa-miR-424-5p
hsa-miR-4745-3p	hsa-miR-664b-3p
hsa-miR-4655-5p	
hsa-miR-543	
hsa-miR-2861	
hsa-miR-1229-5p	
hsa-miR-6850-3p	
hsa-miR-3622a-3p	
hsa-miR-5091	
hsa-miR-1236-3p	
hsa-miR-5007-3p	
hsa-miR-4655-3p	
hsa-miR-7158-5p	
hsa-miR-139-5p	
hsa-miR-652-5p	
hsa-miR-142-3p	
hsa-miR-4793-5p	
hsa-miR-2276-3p	
hsa-miR-4505	
hsa-miR-7846-3p	
hsa-miR-4777-3p	
hsa-miR-4501	
hsa-miR-4738-3p	
hsa-miR-4267	
hsa-miR-4665-5p	

hsa-miR-7702	
hsa-miR-7107-5p	
hsa-miR-4687-3p	
hsa-miR-3189-5p	
hsa-miR-8063	
hsa-miR-3064-5p	
hsa-miR-431-3p	
hsa-miR-7855-5p	
hsa-miR-3155a	
hsa-miR-510-5p	
hsa-miR-127-5p	
hsa-miR-4482-3p	
hsa-miR-4743-3p	
hsa-miR-4722-5p	
hsa-miR-6771-5p	
hsa-miR-639	
hsa-miR-6892-3p	
hsa-miR-6756-5p	
hsa-miR-6880-5p	
hsa-miR-6872-3p	
hsa-miR-6715a-3p	
hsa-miR-330-5p	
hsa-miR-4524a-5p	
hsa-miR-3680-3p	
hsa-miR-3136-5p	
hsa-miR-4647	
hsa-miR-4696	
hsa-miR-7848-3p	
hsa-miR-6758-5p	
hsa-miR-4714-5p	
hsa-miR-4260	
hsa-miR-6777-5p	
hsa-miR-6895-3p	
hsa-miR-6741-5p	
hsa-miR-7108-3p	
hsa-miR-762	
hsa-miR-4684-3p	
hsa-miR-4436b-5p	
hsa-miR-4749-5p	
hsa-miR-3180-3p	
hsa-miR-6812-3p	
hsa-miR-5088-3p	
hsa-miR-3177-3p	
hsa-miR-4304	
hsa-miR-1286	
hsa-miR-6744-5p	
hsa-miR-6125	
hsa-miR-8060	

hsa-miR-1227-5p	
hsa-miR-4732-3p	
hsa-miR-1287-3p	
hsa-miR-3194-5p	
hsa-miR-1471	
hsa-miR-1281	
hsa-miR-647	
hsa-miR-1909-3p	
hsa-miR-618	
hsa-miR-5088-5p	
hsa-miR-6739-3p	
hsa-miR-8056	
hsa-miR-3659	
hsa-miR-648	
hsa-miR-3687	
hsa-miR-28-3p	
hsa-miR-6770-3p	
hsa-miR-7153-3p	
hsa-miR-4532	
hsa-miR-1273g-3p	
hsa-miR-590-5p	
hsa-miR-3610	
hsa-miR-4539	
hsa-miR-4661-5p	
hsa-miR-219b-5p	
hsa-miR-4327	
hsa-miR-551a	
hsa-miR-6843-3p	
hsa-miR-1914-3p	
hsa-miR-6839-5p	
hsa-miR-126-3p	
hsa-miR-1231	
hsa-miR-4687-5p	
hsa-miR-6749-3p	
hsa-miR-3944-3p	
hsa-miR-411-3p	
hsa-miR-4458	
hsa-miR-223-3p	
hsa-miR-1292-3p	
hsa-miR-4322	
hsa-miR-548ba	
hsa-miR-4671-5p	
hsa-miR-6781-5p	
hsa-miR-4685-3p	
hsa-miR-611	
hsa-miR-146b-5p	
hsa-miR-4314	
hsa-miR-4656	

hsa-miR-6801-3p	
hsa-miR-4658	
hsa-miR-1263	
hsa-miR-3127-3p	
hsa-miR-4703-5p	
hsa-miR-5008-3p	
hsa-miR-6765-3p	
hsa-miR-7150	
hsa-miR-7155-3p	
hsa-miR-423-3p	
hsa-miR-4695-5p	
hsa-miR-6869-5p	
hsa-miR-6819-3p	
hsa-miR-4511	
hsa-miR-6778-5p	
hsa-miR-638	
hsa-miR-6126	
hsa-miR-4800-3p	
hsa-miR-8055	
hsa-miR-770-5p	
hsa-miR-3664-5p	
hsa-miR-6719-3p	
hsa-miR-6774-5p	
hsa-miR-296-3p	
hsa-miR-4667-3p	
hsa-miR-6886-3p	
hsa-miR-1180-5p	
hsa-miR-320a	
hsa-miR-34a-5p	
hsa-miR-593-5p	
hsa-miR-6847-5p	
hsa-miR-99b-3p	
hsa-miR-4792	
hsa-miR-4288	
hsa-miR-4734	
hsa-miR-4449	
hsa-miR-4999-5p	
hsa-miR-6860	
hsa-miR-6516-5p	
hsa-miR-6775-5p	
hsa-miR-4781-5p	
hsa-miR-671-3p	
hsa-miR-654-5p	
hsa-miR-6769b-5p	
hsa-miR-6754-3p	
hsa-miR-6724-5p	
hsa-miR-4470	
hsa-miR-6748-5p	

hsa-miR-3150a-3p	
hsa-miR-489-3p	
hsa-miR-892c-3p	
hsa-miR-6090	
hsa-miR-6510-5p	
hsa-miR-6720-3p	
hsa-miR-494-5p	
hsa-miR-661	
hsa-miR-4758-5p	
hsa-miR-6513-5p	
hsa-miR-7114-5p	
hsa-miR-3178	
hsa-miR-500b-3p	
hsa-miR-2115-3p	
hsa-miR-6891-5p	
hsa-miR-4741	
hsa-miR-5002-5p	
hsa-miR-6068	
hsa-miR-636	
hsa-miR-3124-5p	
hsa-miR-4707-3p	
hsa-miR-874-3p	
hsa-miR-4462	
hsa-miR-3190-3p	
hsa-miR-6729-5p	
hsa-miR-564	
hsa-miR-3615	
hsa-miR-3908	
hsa-miR-6082	
hsa-miR-149-3p	
hsa-miR-1200	
hsa-miR-6743-5p	
hsa-miR-3196	
hsa-miR-92a-3p	
hsa-miR-4649-5p	
hsa-miR-3193	
hsa-miR-6814-3p	
hsa-miR-3607-3p	
hsa-miR-3591-5p	
hsa-miR-663b	
hsa-miR-4739	
hsa-miR-193a-5p	
hsa-miR-6816-5p	
hsa-miR-6875-5p	
hsa-miR-4665-3p	
hsa-miR-718	
hsa-miR-320d	
hsa-miR-4797-3p	

hsa-miR-8485	
hsa-miR-1180-3p	
hsa-miR-6836-3p	
hsa-miR-1205	
hsa-miR-6789-5p	
hsa-miR-105-5p	
hsa-miR-6789-3p	
hsa-miR-4252	
hsa-miR-514b-5p	
hsa-miR-4633-3p	
hsa-miR-6722-3p	
hsa-miR-3183	
hsa-miR-4787-5p	
hsa-miR-6751-3p	
hsa-miR-4530	
hsa-miR-508-5p	
hsa-miR-216a-3p	
hsa-miR-1343-5p	
hsa-miR-6764-5p	
hsa-miR-4785	
hsa-miR-6786-5p	
hsa-miR-548as-5p	
hsa-miR-1908-5p	
hsa-miR-520a-3p	
hsa-miR-1268a	
hsa-miR-939-5p	
hsa-miR-7151-5p	
hsa-miR-6765-5p	
hsa-miR-3185	
hsa-miR-4257	
hsa-miR-1228-5p	
hsa-miR-4634	
hsa-miR-6805-5p	
hsa-miR-382-3p	
hsa-miR-1273h-3p	
hsa-miR-7704	
hsa-miR-4707-5p	
hsa-miR-6087	
hsa-miR-3151-3p	
hsa-miR-6753-3p	
hsa-miR-6726-5p	
hsa-miR-572	
hsa-miR-450a-2-3p	
hsa-miR-6089	
hsa-miR-760	
hsa-miR-3944-5p	
hsa-miR-7110-5p	
hsa-miR-4518	

hsa-miR-3153	
hsa-miR-641	
hsa-miR-8069	
hsa-miR-659-3p	
hsa-miR-3665	
hsa-miR-6083	
hsa-miR-433-5p	
hsa-miR-4488	
hsa-miR-148b-3p	
hsa-miR-921	
hsa-miR-2113	
hsa-miR-30d-5p	
hsa-miR-101-3p	
hsa-miR-6785-5p	
hsa-miR-3195	
hsa-miR-30a-3p	
hsa-miR-1469	
hsa-miR-4744	
hsa-miR-4310	
hsa-miR-548j-5p	
hsa-miR-8071	
hsa-miR-663a	
hsa-miR-6777-3p	
hsa-miR-3200-5p	
hsa-miR-668-3p	
hsa-miR-1273c	
hsa-miR-4516	
hsa-miR-6132	
hsa-miR-6738-5p	
hsa-miR-6812-5p	
hsa-miR-3960	
hsa-miR-4435	
hsa-miR-30e-3p	
hsa-miR-4666a-5p	
hsa-miR-3656	
hsa-miR-6873-3p	
hsa-miR-5787	
hsa-miR-410-3p	
hsa-miR-4497	
hsa-miR-3614-3p	
hsa-miR-1268b	
hsa-miR-4466	
hsa-miR-433-3p	
hsa-miR-6736-5p	
hsa-miR-4286	
hsa-miR-4745-5p	
hsa-miR-4638-5p	
hsa-miR-199b-5p	

hsa-miR-4492	
hsa-miR-7853-5p	
hsa-miR-338-3p	
hsa-miR-4281	
hsa-miR-3621	
hsa-miR-370-3p	
hsa-miR-15b-5p	
hsa-miR-5197-3p	
hsa-miR-6803-5p	
hsa-miR-4316	
hsa-miR-383-3p	
hsa-miR-6776-3p	
hsa-miR-4508	
hsa-miR-4674	
hsa-miR-6509-5p	
